# Resuscitation with whole blood or blood components improves survival and lessens the pathophysiological burden of trauma and haemorrhagic shock in a pre-clinical porcine model

**DOI:** 10.1007/s00068-022-02050-6

**Published:** 2022-07-27

**Authors:** Sarah Ann Watts, Jason Edward Smith, Thomas Woolley, Rory Frederick Rickard, Robert Gwyther, Emrys Kirkman

**Affiliations:** 1 Chemical, Biological and Radiological Division, Defence Science and Technology Laboratory, Porton Down, Salisbury, SP4 0JQ UK; 2 Research and Clinical Innovation, Royal Centre for Defence Medicine, Birmingham, B15 2SQ UK

**Keywords:** Trauma, Haemorrhagic shock, Resuscitation, Porcine model, Prolonged care

## Abstract

**Purpose:**

In military trauma, disaster medicine, and casualties injured in remote locations, times to advanced medical and surgical treatment are often prolonged, potentially reducing survival and increasing morbidity. Since resuscitation with blood/blood components improves survival over short pre-surgical times, this study aimed to evaluate the quality of resuscitation afforded by blood/blood products or crystalloid resuscitation over extended ‘pre-hospital’ timelines in a porcine model of militarily relevant traumatic haemorrhagic shock.

**Methods:**

This study underwent local ethical review and was done under the authority of Animals (Scientific Procedures) Act 1986. Forty-five terminally anaesthetised pigs received a soft tissue injury to the right thigh, haemorrhage (30% blood volume and a Grade IV liver injury) and fluid resuscitation initiated 30 min later [Group 1 (no fluid); 2 (0.9% saline); 3 (1:1 packed red blood cells:plasma); 4 (fresh whole blood); or 5 (plasma)]. Fluid (3 ml/kg bolus) was administered during the resuscitation period (maximum duration 450 min) when the systolic blood pressure fell below 80 mmHg. Surviving animals were culled with an overdose of anaesthetic.

**Results:**

Survival time was significantly shorter for Group 1 compared to the other groups (*P* < 0.05). Despite the same triggers for resuscitation when compared to blood/blood components, saline was associated with a shorter survival time (*P* = 0.145), greater pathophysiological burden and significantly greater resuscitation fluid volume (*P* < 0.0001).

**Conclusion:**

When times to advanced medical care are prolonged, resuscitation with blood/blood components is recommended over saline due to the superior quality and stability of resuscitation achieved, which are likely to lead to improved patient outcomes.

**Supplementary Information:**

The online version contains supplementary material available at 10.1007/s00068-022-02050-6.

## Introduction

Military trauma is characterised by life-threatening haemorrhage and tissue injury. The initial treatment priorities to improve patient outcomes are haemorrhage control and prevention and limitation of haemorrhagic shock and its sequelae. For military casualties, current NATO medical doctrine emphasises the importance of timely access to damage control surgery (DCS) and critical care (CC) (the 10.1.2(2) + 2 medical planning guideline) [1]. Recognition that adherence to these timelines may not always be possible, particularly in the context of expeditionary contingency operations, has given rise to the concept of ‘prolonged casualty care’, defined as the application of techniques to sustain a casualty, and maximise their chances of survival, if any component of 10.1.2(2) + 2 is likely to be exceeded) [1].

The burden associated with the delivery of clinical care over extended timelines is increased personnel, logistics and equipment [2]. In the pre-hospital environment this burden may be partially reduced with the forward deployment of blood/blood products as they as they have been shown to reduce mortality. Although it seems that there is a preponderance of published opinion in one direction, opinion publications seem to outnumber primary data publications, and there is no universal agreement between primary data publications, even amongst the most recent papers. Brill et al. [3] concluded that treatment with whole blood conferred a survival benefit compared to those given fractionated blood (red cells, plasma and platelets) and Guyette et al. [4] concluded that resuscitation with combined red cells and plasma resulted in higher survival compared to that seen in patients resuscitated with crystalloid. By contrast, Crombie et al. [5] found no superiority of combined red cells and plasma when compared to crystalloid with respect to survival or lactate clearance. The evidence supporting pre-hospital blood is limited to short pre-hospital times of < 90 min only [6–9]. A ‘forward blood’ capability is not without its own logistical burden, potentially hindering its implementation. Lyophilised plasma and crystalloids (the latter still standard care in many countries in both civilian and military practice) offer potential solutions to logistic and equipment constraints and whole blood in the pre-hospital setting is gaining momentum [10, 11], and for the military this could be facilitated by both a cold-stored whole blood as well as a walking blood bank capability.

Although blood/blood products may form part of the solution for the increased burden of prolonged casualty care to both care providers and the patient, a knowledge gap remains with regard to their efficacy in this context especially with finite resources. Therefore, the aim of this study was to evaluate the quality of resuscitation afforded by blood/blood products or crystalloid resuscitation in a porcine model of militarily relevant traumatic haemorrhagic shock (THS) in the context of extended times to DCS and/or CC.

## Materials and methods

This study underwent local ethical review and was carried out under the authority of Animals (Scientific Procedures) Act 1986. The study used a terminally anaesthetised porcine THS model (combined tissue injury and haemorrhage).

### Study design and blinding

This was a prospective randomised study in juvenile female cross-bred Large White pigs. Each animal represented one experimental unit. The study was conducted under terminal anaesthesia, and was terminated with an overdose of sodium pentobarbitone (Euthatal, Merial Animal Health Ltd, Harlow, UK).

Animals were randomised (Excel Random Number Function) to one of five experimental groups: Group 1 = no treatment; Group 2 = 0.9% NaCl; Group 3 = PRBC:FFP (FFP = fresh frozen plasma; Group 4 = FWB (FWB = fresh whole blood); and Group 5 = FFP. Details of the sample size calculation are given in Supplemental Digital Content 1.

It was not possible to blind the researchers to treatment.

### Blood bank

Blood was collected by exsanguination from terminally anaesthetised female Large White pigs and prepared, stored and used as previously described [12] in accordance with UK Guidelines [13]. Fresh whole blood was stored unfiltered at 22 °C ± 2 °C and used the following day. Prior to use, all donor products were forward and reverse matched to recipient blood. In addition, since PRBC and FFP from different donors were used for resuscitation, they were also cross-matched with each other. If a cross-reaction occurred, animals were re-assigned to another group according to the randomisation. This was considered a random event not influenced by human judgement.

### Experimental procedures

Anaesthesia and surgical instrumentation are described in full online (see text, Supplemental Digital Content 1).

### Anaesthesia

Oral intake was withheld overnight but animals had free access to water. Animals were sedated with intramuscular midazolam (0.1 mg/kg) and anaesthesia was induced and maintained with isoflurane in oxygen and nitrous oxide. Following intubation animals were ventilated to maintain normocapnia with tidal volume set to a maximum of 8 ml/kg.

Once venous access was established, anaesthesia was converted from isoflurane to a constant intravenous infusion of alfaxalone. Depth of anaesthesia was assessed using palpebral and pedal withdrawal reflexes. Body temperature was maintained at approximately 38 °C throughout the experiment using external heating/cooling and blankets as appropriate.

### Instrumentation

Surgical sites were aseptically prepared using povidone-iodine solution. Intravascular cannulation allowed cardiopulmonary monitoring, intermittent blood sampling and the administration of substances. A balloon-tipped flow-directed cannula was placed in the pulmonary artery.

An infusion of 0.9% NaCl, 10 ml/kg, started once venous access was established and stopped on completion of the instrumentation. Splenectomy, bladder cannulation and placement of a snare around the left medial lobe of the liver was performed via a midline laparotomy.

Animals were weaned off the ventilator and breathed spontaneously for the remainder of the study. Pressure-controlled synchronised intermittent mandatory ventilation (SIMV) was initiated if marked respiratory depression occurred during the injury and resuscitation phases.

#### Experimental protocol

**Fig. 1:**
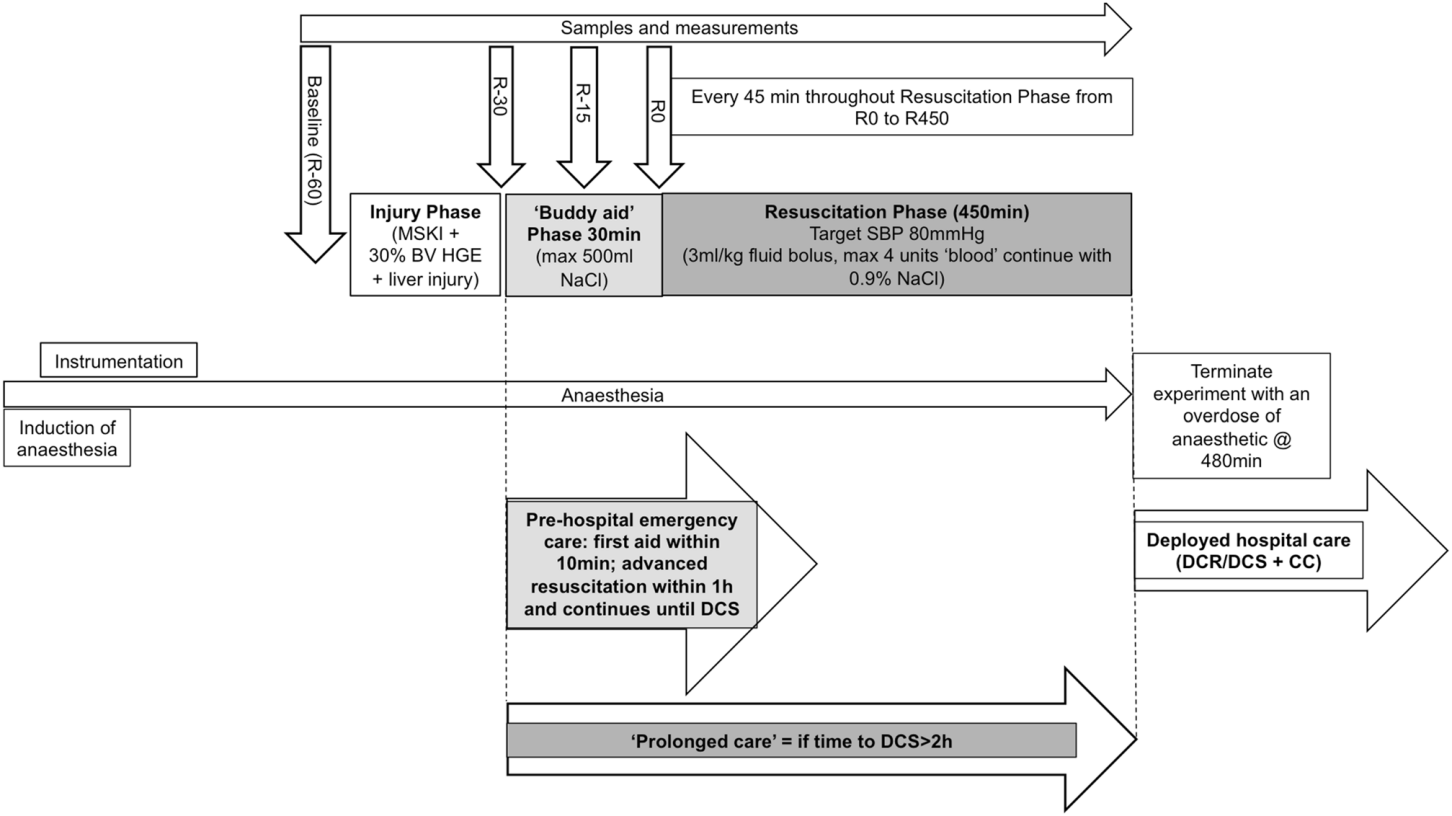
A time line of the experimental protocol including blood sample time points. ‘Prolonged care’ as applied to this experimental study is defined as a delay to DCS beyond the 2 h defined in NATO medical planning guidelines 1. “Buddy Aid” Phase of 30 min, resuscitation phase of 450 min and termination of experiment at 480 min from the onset of resuscitation. *DCR *damage control resuscitation, *DCS* damage control surgery, *CC *critical care

A timeline for the study and how the study is aligned to the concept of ‘prolonged casualty care’ is shown in Fig. 1.

##### Baseline

Baseline measurements were taken 60 min post-instrumentation.

##### Injury phase

A soft tissue injury (4 shots to the right thigh) was created using a captive bolt (Cash Special, Accles and Shelvoke, Sutton Coldfield, UK). Two minutes later animals underwent a controlled haemorrhage (30% blood volume) at an exponentially reducing rate, over 9 min and 41 s as previously described [14]. Once completed, the snare around the liver lobe was pulled to initiate an uncontrolled incompressible haemorrhage.

##### ‘Buddy aid’ (shock) phase (30 min)

If SBP fell below 60 mmHg 0.9% saline (200 ml/min) was administered until 60 mmHg attained, to a maximum of 500 ml. Additional blood was removed (maximum 10% original blood volume) according to a combination of SBP, cardiac output (CO) and blood biochemistry (lactate and arterial actual base excess (ABE)) to ensure a consistent degree of shock across animals and groups.

##### Forward prolonged resuscitation (450 min)

Fluid was administered via a Belmont Rapid Infuser (Belmont Instrument Corporation, US) except for Group 4 where whole blood was administered via PHD ULTRA™ infusion pump (Harvard Apparatus, Cambridge UK) (the effect of the Belmont on pig platelet function is unknown).

Group 1: No fluid resuscitation.

Groups 2–5: The trigger for a 3 ml/kg bolus of fluid (200 ml/min) was SBP < 80 mmHg. The maximum number of blood product units given to any one animal was 4, thereafter resuscitation continued with 0.9% saline. FWB (4 units), PRBC:FFP (4 units of each) and FFP (4 units).

##### Study end

The study endpoint was 450 min from the onset of resuscitation.

### Cardiopulmonary monitoring

Temperature, end-tidal CO_2_, ECG and pulse oximetry (Propaq CS, WelchAllyn, Aston Abbotts, UK) were monitored throughout. Continuous systemic arterial blood pressures were measured using strain gauge manometers (Sensonor 840, SensoNor a.s., Norway) with zero pressure for all transducers set at heart level. A flow-directed balloon-tipped flotation catheter was also used to determine CO by thermodilution as a 6 min rolling average (Vigilance Volumetrics CEDV, Edwards Lifesciences, US). Blood pressures, heart rate (HR), and CO were recorded using a computerised data acquisition system (Maclab 8/s, ADInstruments, UK) and associated software (Chart v4.2.3, ADInstruments).

### Blood sample analysis

Samples for arterial and mixed venous blood gas analysis and haematology were collected at pre-defined time points, see Fig. 1.

### Inclusion and exclusion criteria

Animals were included in the analysis if SBP ≥ 50 mmHg at the onset of resuscitation and they received at least 1 fluid bolus during the resuscitation phase. Animals failing to meet these criteria were excluded.

### Outcomes

The primary outcome of the study was survival. Secondary outcomes were compared between Groups 2–5 only and included physiological status, in particular the degree of shock, and volume of fluid administered.

### Statistical analysis

Survival times were compared using Kaplan–Meier plots and logrank test, followed by planned between group comparisons using Mantel–Haenszel probability levels (NCSS Version 11 statistical package, LLC, East Kaysville, Utah). Discrete data (number of boluses of fluid) were assessed using the Kruskal–Wallis analysis of variance, followed where appropriate by a planned between group multiple comparison. All other data were assessed for normality and subjected to transformation if necessary. Single time-point analyses were made using 1-way ANOVA with Fisher comparison of means for planned between group comparisons (NCSS Version 11), unless indicated otherwise. Data with repeated measures were analysed by linear mixed model ANOVA with repeated measures over time, using pre-injury baseline values as the covariate for the injury/shock phase analysis, and the end-shock value as the covariate for the resuscitation phase using the R Statistics Package (R Studio v1.2.1335, Boston, Massachusetts), with planned comparisons between groups as indicated. P < 0.05 was taken as statistically significant. All data are presented as mean ± SEM unless indicated otherwise.

## Results

### Inclusion and exclusion

Fifty-six animals were randomised to the study. Eight animals failed to meet the inclusion criteria, leaving 48 animals that met the inclusion criteria. To prevent skewing in favour of ‘fluid resuscitation’ versus no treatment the following were excluded from the data analysis: one animal each from Groups 2 and 3 did not develop shock post-injury and required minimal fluid resuscitation (outliers from their respective groups); and 1 animal from Group 1 had a blood potassium ion concentration of 8.8 mM at the start of the resuscitation and died in less than 5 min leaving a total of 45 animals (9 per group).

### Baselines

Baseline values for key cardiovascular, respiratory and oxygen transport parameters were within the ranges expected for anaesthetised pigs (see Supplemental Digital Content 2 Table 1). There were no significant differences between groups except for lactate, where there was a statistically significant difference between groups due solely to the FWB group being slightly higher than the others, but this difference is unlikely to be of clinical consequence.

### Injury and shock phase

Tissue injury, progressive haemorrhage and the subsequent 30 min shock phase led to cardiovascular compromise and the development of a shock state, with the expected significant falls in oxygen delivery, consumption and arterial base excess (ABE), but no significant difference between groups. (For detail and statistical P values, see Supplemental Digital Content 3 Table 2).

The no treatment group was excluded from further analysis, except for survival times, as the short survival time made secondary outcome comparisons meaningless.

### Effects of prolonged hypotensive resuscitation

(For statistical *P* values, see Supplemental Digital Content 3 Tables 2–4.)

#### Resuscitation fluid volume and physiological responses

##### Fluid volume

**Fig. 2:**
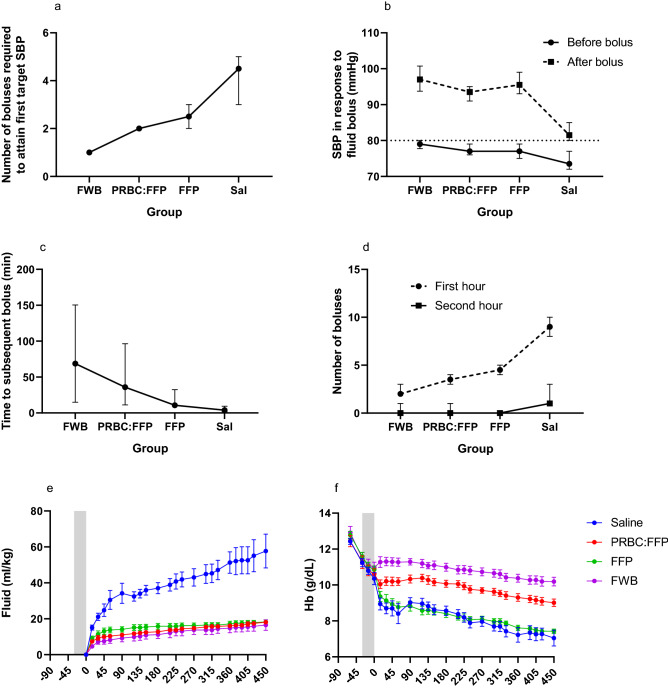
Quantity and effect of fluid resuscitation. **A** Number of boluses given from R0 to attain target SBP; **B** SBP before and after the following fluid bolus; **C** time to the subsequent bolus; **D** number of boluses given in the first and second hour from the onset of the resuscitation phase; **E** cumulative fluid total during the resuscitation phase; **F** arterial haemoglobin concentration

There were no significant differences between groups in the volume of saline administered to maintain SBP at a minimum of 60 mmHg during the Shock Phase. The cumulative volume of fluid given for resuscitation, however, was significantly different between groups (Fig. 2E). Overall, the Saline group was significantly different to each of the groups given blood/blood products (*P* < 0.0001 in each case), with an approximate threefold greater volume for saline. Differences between the other groups were relatively minor (see Supplemental Digital Content 3).

##### Physiological response: initial response to fluid resuscitation

At the end of the shock phase, there was no significant difference in SBP between groups (see Injury and shock phase). SBP was below the resuscitation trigger of 80 mmHg in 8/9 animals in each of the FWB, PRBC:FFP and FFP groups and 9/9 animals in the Saline group. There was no significant difference between groups in the time to initiation of fluid administration (Supplemental Digital Content 4). There was, however, a significant difference between groups in the number of boluses required to attain or exceed the resuscitation trigger (Fig. 2A).

##### Physiological response: cardiovascular stability during the resuscitation phase

Although there was no significant difference between groups in the SBP before initiation of the following bolus (after attaining or exceeding the resuscitation trigger, 80 mmHg), there was a significant difference between groups in SBP seen 30 s after the end of bolus administration (Fig. 2B), which was due to the SBP being significantly lower in the Saline group compared to all of the others (*P* < 0.006 in each case). The time to the subsequent bolus was also different between groups (Fig. 2C), with FWB being significantly longer than saline (*P* < 0.05), and the other groups being intermediate. Finally, the number of boluses given in response to a fall in SBP below the resuscitation trigger during the first and second hours of resuscitation was significantly different between groups (Fig. 2D), with the Saline group requiring the most boluses.

**Fig. 3:**
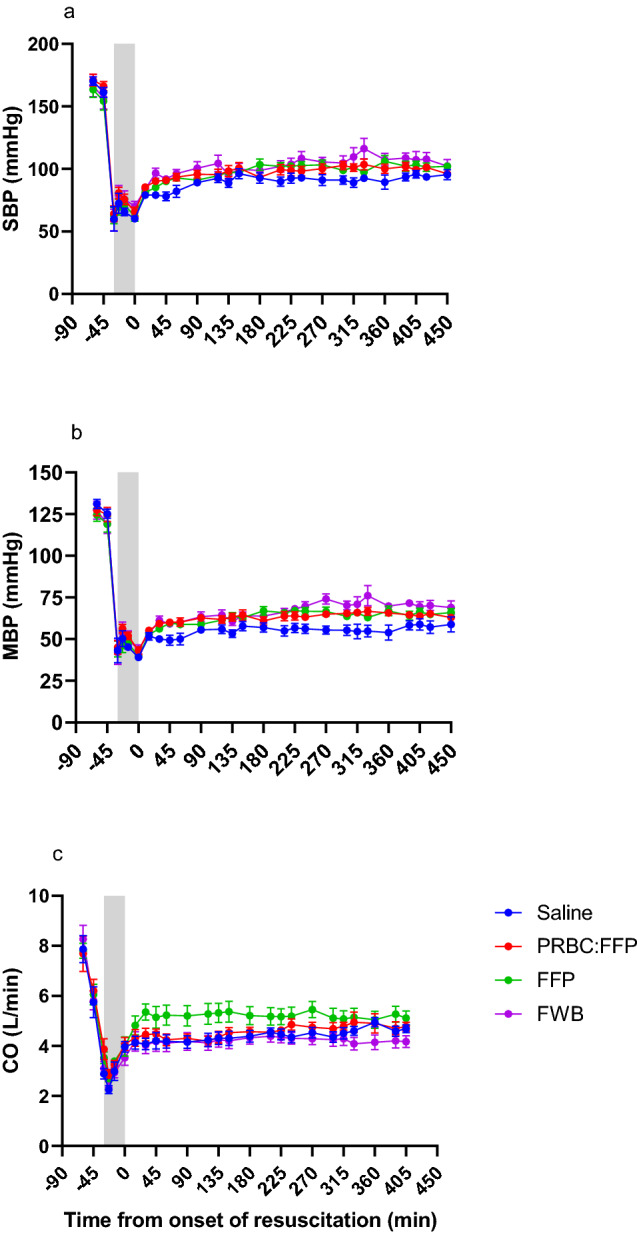
Haemodynamic changes from baseline to the end of the resuscitation phase (450min) with the grey-shaded area representing the shock phase. **A** systolic blood pressure (SBP); **B** mean arterial blood pressure (MBP); and **C** cardiac output (CO)

Overall fluid resuscitation, given in response to a fall in SBP below the trigger (80 mmHg), ensured SBP remained above the trigger throughout the study (Fig. 3A); however, the Saline group showed the lowest blood pressure. Concurrent with the increased blood pressure there was also a significant increase in CO in all groups, although again there were differences between groups. Notably, the group given FFP displayed the highest CO between the first and third hours of resuscitation (*P* < 0.04, Fig. 3C).

#### Oxygen transport

**Fig. 4:**
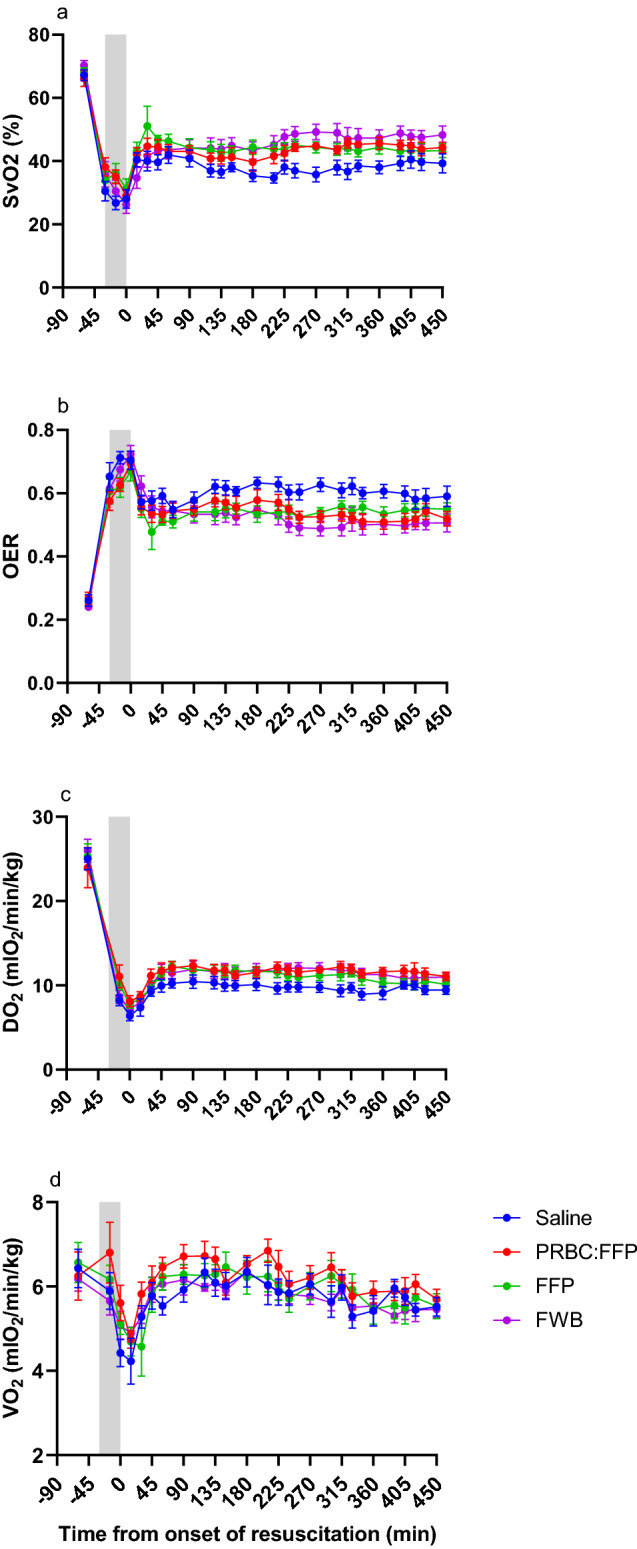
Oxygen transport from baseline to the end of the resuscitation phase (450min) with the grey-shaded area representing the shock phase. **A** Mixed venous oxygen saturation; **B** oxygen extraction ratio (OER); **C** oxygen delivery (DO_2_); and **D** oxygen consumption (VO_2_)

Fluid resuscitation was associated with a significant and sustained increase in venous oxygen saturation (SvO_2_) (Fig. 4A). There was a significant difference in pattern of response between groups, and eventually the group resuscitated with 0.9% saline displayed the lowest SvO_2_. Arterial oxygen saturation (SaO_2_) was well maintained in all groups during the resuscitation period. There were significant difference between groups in arterial oxygen content (Supplemental Digital Content 5), with the Saline and FFP groups similar and lowest (*P* = 0.9196) during resuscitation. Oxygen delivery (DO_2_) increased significantly during resuscitation, although the differences between groups were not statistically significant, saline showed the smallest increase while FFP was amongst the other blood groups (Fig. 4C). Concurrent with the improved haemodynamics, oxygen extraction ratio (OER) fell from the ceiling attained during the shock phase in all groups. Of note, OER was highest in the group resuscitated with 0.9% saline (Fig. 4B). Oxygen consumption (VO_2_) increased significantly from the nadir seen in the shock phase, without differences between groups (Fig. 4D).

#### Biochemical changes

**Fig. 5:**
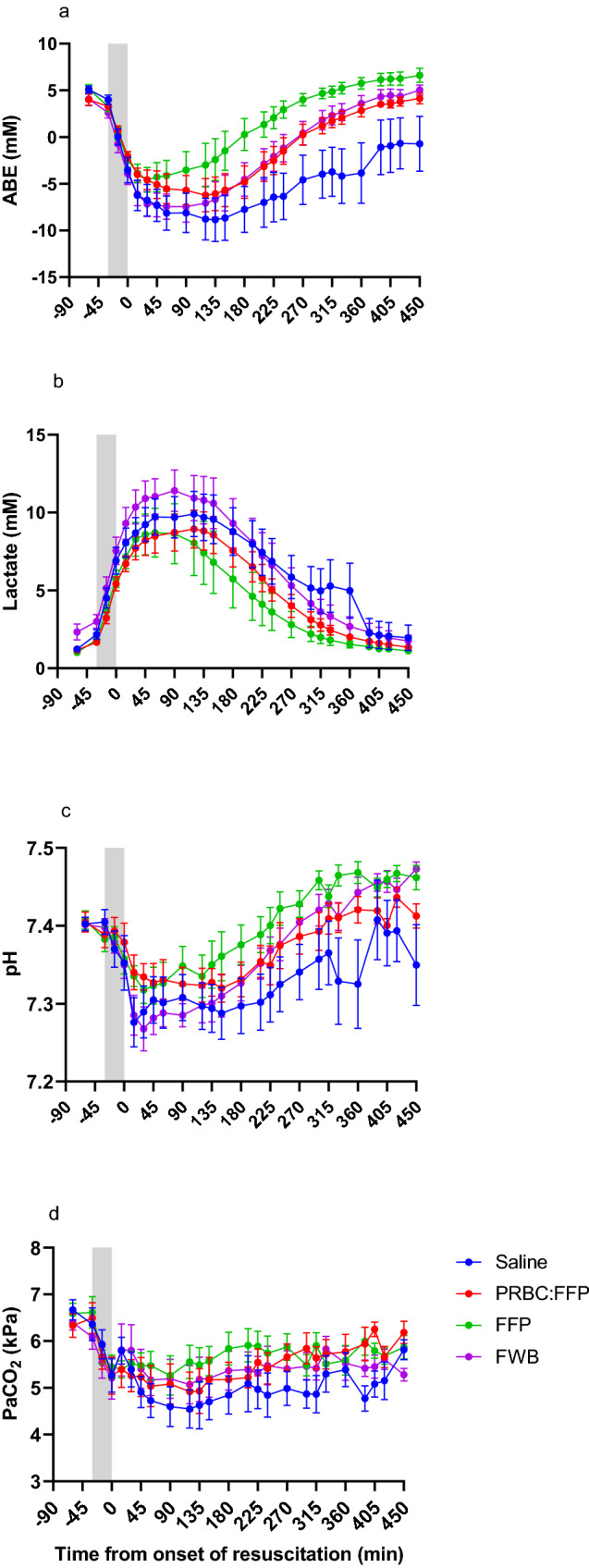
Arterial blood biochemistry and degree of acidosis from baseline to the end of the resuscitation phase (450min) with the grey-shaded area representing the shock phase. **A** Actual base excess (ABE); **B** lactate; **C** pH; and **D** arterial partial pressure of carbon dioxide (PaCO_2_)

Evidence of the metabolic acidosis with partial respiratory compensation is shown in Fig. 5A–D (described in Supplemental Digital Content 3), hyperchloraemic acidosis was evident in the Saline group (Supplemental Digital Content 6). As expected arterial lactate continued to rise and ABE continued to decline during the early resuscitation phase, before the trends were reversed after approximately 90 min of resuscitation. There were clear differences between groups, and for both parameters there were significant changes over time and significant differences in pattern of response and absolute values between groups (except for the difference between groups in lactate levels) (Fig. 5A). Saline group displayed the lowest ABE throughout the resuscitation period, and was significantly different to both FWB and FFP (*P* < 0.005).

Finally, resuscitation was associated with significant changes in arterial haemoglobin (Hb) levels. There were clear differences in pattern of response and absolute levels between groups (Fig. 2F), which fell into the following rank order of Hb levels: FWB > PRBC:FFP > FFP = Saline). Despite this, DO_2_ was higher in the FFP group compared to the Saline group, and not different to the other blood products.

### Survival

**Fig. 6:**
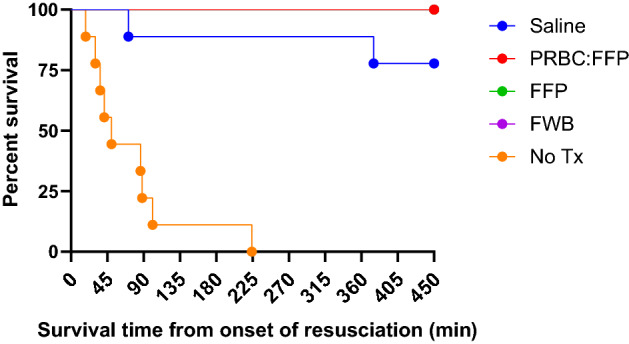
Kaplan–Meier survival curve showing the survival time from the onset of resuscitation. No Tx = No treatment group. There were no deaths in the blood/blood products groups and the survival curves overlap

The Kaplan–Meier plot showing survival in each of the groups is shown in Fig. 6. There were significant differences in survival time between groups. None of the animals in the no treatment group survived beyond 224 min from the onset of resuscitation (7/9 died within the first 90 min). By contrast, all the animals in the groups given blood/blood products and 7/9 animals resuscitated with 0.9% saline survived to the end of the study (the remaining two died at 71 and 375 min). Planned further analysis indicated that survival time in the no treatment group was significantly shorter than each of the other groups and although there was a suggestion that survival time was shorter with saline than the blood product groups this did not attain statistical significance. However, when Groups 3 (PRBC + FFP) and 4 (FWB) were combined to form a “blood group” (increasing the power of the analysis), a significant difference was apparent between the saline and the “blood group”.

## Discussion

The purpose of this study was to evaluate the pathophysiological burden of THS and hypotensive resuscitation in the context of extended times to DCS and/or CC. The results show that fluid resuscitation, with either blood/blood components (PRBC:FFP, FWB or FFP) or crystalloid (0.9% saline), administered in response to the same clinical trigger (SBP < 80 mmHg) significantly increased survival compared to no fluid resuscitation, supporting earlier reports [15, 16]. Even though the volume of fluid administered was approximately three times less with blood/blood components compared to saline the quality of resuscitation was better, with improved haemodynamics and less shock.

### Clinical guidelines and planning

Haemorrhage is the leading cause of preventable trauma death [17, 18] and clinical guidelines recommend interventions that limit blood loss, including permissive hypotensive resuscitation, as well as timely DCS and definitive care [19–23]. There has been a recent shift, with an acknowledgement that strict adherence to hypotensive resuscitation may be unnecessary particularly with the increasing use of blood/blood products [24]. Any benefit of hypotensive resuscitation in a hypovolaemic patient may be lost with extended times to DCS and/or CC, as it may exacerbate tissue hypoperfusion, worsening shock, as seen in models of haemorrhagic shock with and without blast [25], and leading to sequelae such as blood failure [26]. Data from this study demonstrate that permissive hypotensive resuscitation, with fluid boluses given only when SBP reaches the trigger to treat point (80 mmHg), results in attainment of higher blood pressures which are attained for longer with blood/blood products compared to saline.

The findings of this study are consistent with some of, and significantly add to, the evidence that is already published from pre-clinical studies [27–29]. A significant body of studies addressing cellular injury and inflammatory responses initiated by resuscitation was reviewed by Rhee et al. in 2003 [16]. The review [16] included approximately eight animal studies in pigs and rats that principally utilised models of substantial haemorrhage and shock without additional injury (other than essential surgery), and relatively short resuscitation periods (usually 1–4 h), as well as studies on human blood. Although the studies focussed on the effects of crystalloids, principally Ringer’s lactate, a number had whole blood and plasma as control groups. A very important finding from the review was the concept of a “resuscitation injury” which is distinct from “reperfusion injury” [16]. The review [16] concluded that resuscitation with hypotonic/isotonic crystalloids and artificial colloids elicit severe immune activation and upregulation of cellular injury mechanisms, an effect not seen with natural colloids (albumin), plasma or fresh whole blood [16]. Fresh whole blood was viewed as being “by far the best and most effective fluid for resuscitation of hemorrhagic shock” [16], although at the time it was not clinically available. This conclusion has to be interpreted in the context of a short resuscitation period, and endpoints of inflammation and tissue damage. It was, therefore, unclear whether the benefit would persist with substantially longer hypotensive periods, for example, when prolonged evacuation timelines are being mimicked.

There are several studies that compare some individual treatment elements (fluids), for example plasma versus crystalloid or PRBC:FFP vs crystalloid, very few have compared all the treatment modalities presented currently in one study. Consequently, comparisons are made by extrapolation between studies. One notable study that can be compared to the study reported in this manuscript examined the effects of resuscitation with a range of fluids including fresh whole blood (warm < 24 h), PRBC:FFP, plasma and crystalloid over a 5 h post-resuscitation period [29]. The primary focus of that study was re-bleeding and coagulation and all the fluid was given in the first hour mimicking a different clinical scenario. The changes in arterial base excess were small in comparison to this current study where there were marked changes with significant difference between groups. Although both models include a substantial haemorrhage, the model reported by Sondeen et al. [29] did not include any significant soft tissue trauma, which is widely recognised as an important feature of models of trauma and resuscitation [12, 30–32]. Indeed, Majde [32] goes further to emphasise that pre-clinical models of trauma in particular should simulate the treatments that will be experienced on the battlefield or rural setting (including time delays in treatment and degree and duration of hypotension) if that is the scenario that they are addressing [32].

Holcomb et al. [33] emphasised the importance of early in-hospital treatment with plasma and red blood cells (and minimising the use of crystalloids) in casualties requiring massive transfusion based on anecdotal accounts of casualty treatment and review of the literature, echoing similar conclusions based on clinical observations in earlier conflicts [34]. Recent systematic reviews highlight there is still much uncertainty regarding the optimal timing and composition of blood product use in patients with major haemorrhage [35–40]. This may be due, in part, to the different blood products used, in particular whole blood, for example cold-stored low titre O negative whole blood, leukoreduced whole blood with added apheresis platelets and warm fresh whole blood have all be used. In 2018, Shand et al. commented that “Further research specific to pre-hospital practice is required to guide the development of evidence-based protocols” [38]. Rijnhout et al. [39] found that pre-hospital resuscitation with combined red cells and plasma did reduce 30 day, but not 24 h mortality. Crowe et al. [36] also concluded that “compared with conventional component transfusion, whole blood was not associated with 24-h or in-hospital mortality”. But went on to say that “there were important limitations with and heterogeneity among the primary studies. Additional study is needed to determine the effectiveness of whole blood” [36], a sentiment echoed by Cruciani et al. [40]. Avery et al. [35] concluded that they were unable to determine whether whole blood compared to component therapy improved 30-day survival due to the poor quality of evidence available. Malkin et al. [37] in their review of whole blood versus blood components stated “The quality of existing data is poor and further studies are required”. Even with the evidence from RCTs and opinion that there is improved survival with pre-hospital blood product resuscitation compared to crystalloid actual blood/blood product usage in the pre-hospital setting is low [41].

Logistical constraints, associated with deployed military medical services, could influence the availability of resuscitation fluids. Although saline resuscitation did reverse the shock that developed post-injury the physiological response with saline was inferior to the groups given blood/blood components, and significantly greater volumes of fluid were required. Plasma products potentially offer logistical advantages over red blood cell containing products and we have shown that the physiological response in the group resuscitated with FFP (as an example plasma product) was at least as good as that seen in the groups resuscitated with red cells (FWB and PRBC:FFP). It is unlikely, therefore, that lack of oxygen-carrying capacity was the primary cause of the poorer outcomes in the Saline group.

### Response to fluid resuscitation

The response to resuscitation was much more stable in the groups given blood/blood products compared to saline, not only with respect to the overall amount of fluid (and, therefore, boluses given) but also with respect to the length of time between boluses and the rapidity with which blood pressure fell once a bolus had been given, therefore, reducing the physical as well as mental burden of the care provider. In our controlled experimental setting, a team member was designated to monitor blood pressure almost continuously (certainly with no more than 30 s gaps) to initiate a bolus of fluid once SBP fell convincingly below 80 mmHg (overall SBP in the range 75–80 mmHg for 30 s, or SBP < 75 mmHg). Even in the time taken to process this information, reach a decision and initiate a bolus, a trend of a lower blood pressure before initiation of fluid administration was seen in the Saline group compared to the others (Fig. 2B). This need for constant monitoring with saline resuscitation is likely to consume a significant amount of ‘clinical bandwidth’ in a small team managing a casualty. The stability provided by the blood/blood products is likely due to oncotic pressures exerted by plasma proteins. Overall, a far greater volume of fluid was administered to the Saline group, which was both clinically and statistically significant, and in line with clinical evidence that greater volumes of crystalloid than colloid are required to achieve the same target [42]. Interestingly, amongst the blood/blood product groups, more FFP was initially required, although this difference was relatively small, and attained statistical significance only in comparison with FWB, possibly due to FFP being hypo-oncotic [43]. This difference had disappeared by the end of the study.

Alongside the rise in SBP following fluid administration, there was a concomitant increase in DO_2_, of sufficient magnitude to allow OER to fall and VO_2_ to increase, with a fall in lactate and base deficit consistent with beginning to reverse the shock [44]. The effect was similar in blood/blood product groups, but smallest in the group given saline, most likely as the increased CO was tempered by a fall in haemoglobin levels. Unlike the current study, Leong et al. [45] found that crystalloid resuscitation (single 500 ml bolus) did not reverse shock. The FFP group, which also showed a fall in haemoglobin levels, mounted the biggest increase in CO and, in agreement with others [29], DO_2_ in this group was similar to that seen in FWB and PRBC:FFP groups. In contrast to saline, FFP may limit shock and its sequelae via improved rheology of the blood (although the situation here is complex [46] and the view is not universally accepted [47]), as well as the reported positive effects on the endothelium [48–51].

### Limitations

The results seen following saline resuscitation may be surprising to some who may question the severity/applicability of the model. Our model of THS, however, led to the development of shock by the end of the Shock Phase and is consistent with other models of THS [29, 45, 52–54]. All groups were hypotensive and had significant depression of cardiovascular function, oxygen delivery and oxygen consumption, despite an increase in oxygen extraction ratio to maximal levels (approximately 70–75%). The developing metabolic acidosis (Fig. 5) together with the haemodynamic and oxygen transport changes (Figs. 3 and 4) clearly indicated shock [44]. As a measure of the severity of the model, there was continued depression of oxygen delivery and consumption and worsening of shock in Group 1 (no fluid), with death in half the animals by 60 min and all animals by 4 h.

Haemodynamic/oxygen transport responses to trauma and resuscitation in pigs are representative of humans [31, 55]; consequently, the pig is used extensively by other groups in this context [56].

The study was conducted under anaesthesia and the effects of prolonged anaesthesia on the model were not assessed. The anaesthetic regime chosen preserves the cardiovascular reflex responses to injury and the visceral alerting response of the defence reaction [57], therefore, is more representative of the conscious human than other anaesthetic agents. Any effects of anaesthesia would be the same in all groups, thus between group comparisons remain valid.

The study was designed to represent prolonged pre-hospital times, but it still only examined the first few hours post-injury, and long-term sequelae were not examined. This is particularly important in the context of saline resuscitation where the detrimental effects, such as tissue oedema and inflammation may manifest later [58, 59].

Although the liver injury had the potential to continue to bleed throughout the experiment minimal free blood was present in the abdomen (Supplemental Digital Content 7), indicating the model to be one where the patient has bled (bleeding controlled, for example with the use of a tourniquet). Any detrimental effects associated with oxygen-carrying capacity are more likely to be seen when there is continued haemorrhage, which is being addressed in a subsequent study. We chose to administer a maximum of 4 units of blood/blood product, equating to approximately 1800 ml for FWB, 2240 ml for PRBC:FFP and 1120 ml FFP potentially biasing the results against FFP. This was not the case as there were no differences in the volume of fluid administered to those groups and, no animal in these groups required additional saline.

In conclusion, we have shown that resuscitation with blood/blood components, initiated in response to hypotension, improves pathophysiological outcomes compared to saline. The mostly likely explanation is that blood/blood components limit and/or treat the oxygen debt (with likely knock-on effects of reduced endothelial dysfunction and coagulopathy). Therefore, in order to maximise survivability, particularly in the context of prolonged casualty care, fluid resuscitation should be initiated as early as possible, preferably with blood/blood products, as saline was inferior to blood/products with a greater burden of shock, an approximately threefold fluid volume burden, and a much less ‘stable’ resuscitation likely to consume more clinical bandwidth to both monitor and administer fluid to the casualty.

© Crown copyright (Dstl and MOD) 2022. This material is licensed under the terms of the Open Government Licence except where otherwise stated. To view this licence, visit http://www.nationalarchives.gov.uk/doc/open-government-licence/version/3 or write to the Information Policy Team, The National Archives, Kew, London TW9 4DU, or email: psi@nationalarchives.gsi.gov.uk”.

## Supplementary Information

Below is the link to the electronic supplementary material.

Supplementary file1 (PDF 110 KB)

Supplementary file2 (PDF 44 KB)

Supplementary file3 (PDF 80 KB)

Supplementary file4 (PDF 113 KB)

Supplementary file5 (PDF 191 KB)

Supplementary file6 (PDF 632 KB)

Supplementary file7 (PDF 50 KB)
