# Supplementary material for: Resuscitation with whole blood or blood components improves survival and lessens the pathophysiological burden of trauma and haemorrhagic shock in a pre-clinical porcine model
Source: Eur J Trauma Emerg Surg. 2022 Jul 27;49(1):227–39. doi: 10.1007/s00068-022-02050-6 (PMC9925484; doi:10.1007/s00068-022-02050-6)
Supplement: Supplementary file 1 — Supplementary file1 (PDF 110 KB) [file 68_2022_2050_MOESM1_ESM.pdf]

## Methods

Ethical Statement

The study was performed under the authority of the UK Animals (Scientific Procedures) Act 1986. The work received institutional ethical approval and a UK Home Office licence was granted. The work was conducted in accordance with the Guidance on the Animals Scientific Procedures Act (1986) and the Code of Practice for the housing and care of animals bred, supplied or used for scientific purposes.

Animals

Animals were purchased from a UK Commercial supplier a minimum of 7 days prior to experimental procedures. Animals were housed indoors, fed *ad libitum* on a complete diet comprising course ground mixture of wheat, barley, soya protein, vitamins and minerals and had free access to water.

Study Design

Groups 1 and 2 were shared with another study (to be reported in a subsequent manuscript), to give a total of 6 groups that were fully randomised (a total of 5 groups reported here).

*Blood Bank*

Blood was collected by exsanguination from terminally anaesthetised female Large White pigs as previously described by this group[1]. Briefly standard units of blood were collected from a carotid cannula at a flow rate of 65-90 ml/min into each CPD blood collection bag (RCB434CCL, Haemonetics, UK). The blood was processed according to standard UK blood transfusion protocols[2]. The resulting units of packed red cells (PRBC) were stored at 4°C (LabCold Blood Bank, UK) and used within 14 days of collection. The plasma (FFP) was

[1] Watts S, et al. Evaluation of Prehospital Blood Products to Attenuate Acute Coagulopathy of Trauma in a Model of Severe Injury and Shock in Anesthetized Pigs. Shock. 2015 Aug;44 Suppl 1(Suppl 1):138-48.
[2] Joint United Kingdom (UK) Blood Transfusion and Tissue Transplantation Services Professional Advisory Committee. Guidelines for the Blood Transfusion Services in the UK 8th Edition. 2013.

fast-frozen (MP1100 Plasma Freezer System, Thermogenesis, US), stored at -30°C (LabCold Plasma Freezer, LabCold, UK) and used within 6 months of collection.

Fresh whole blood was stored unfiltered at 22°C±2°C following collection and used the following day.

FFP was thawed at 37°C in a dry plasma thawer (Sahara III Maxitherm, Sarstedt, Germany) immediately before use.

Prior to use all donor products were forward and reverse matched to recipient blood. In addition, since PRBC and FFP from different donors were used for resuscitation, they were also cross-matched with each other. For cross matching, red cells were washed three times in 0.9% saline by sequential addition of saline, centrifugation and removal of supernatant, culminating in a 1:10 dilution of the original red cell concentration in 0.9% saline. The red cell suspension and plasma for cross matching were then mixed in a 1:1 proportion and incubated for 15 min at room temperature, centrifuged and checked for agglutination and gross haemolysis. In cases of doubt (without clear agglutination) the samples were re-mixed and incubated at 38.5°C for a further 15 min, re-centrifuged and checked for agglutination. Only samples with clear absence of agglutination were used for transfusion. On initiation of transfusion the animals were closely monitored for recognised clinical signs of a transfusion reaction[34]. No transfusion reactions were observed in this study.

Experimental procedures

*Instrumentation*

Intramuscular midazolam hydrochloride (Hypnovel, Roche Products Ltd, Welwyn Garden City, UK) (0.1mg/kg) was administered 15 minutes prior to induction of anaesthesia with isoflurane (Isoflurane-Vet, Meriel Animal Health Ltd, Bracknell, UK) in oxygen and nitrous

[3] Tinegate, H et al. Guidelines on the investigation and management of acute transfusion reactions. Prepared by the BCSH Blood Transfusion Task Force. British Journal of Haematology. 2012 159 143-153

[4] Payandeh, M et al. Descriptions of acute transfusion reactions in the teaching hospitals of Kermanshah University of Medical Sciences, Iran. International Journal of Hematology – Oncology and Stem Cell Research. 2013 7 (2) 11-16

oxide (50:50). Animals were placed supine and following endotracheal intubation surgical anaesthesia was maintained with isoflurane (1-2%) in oxygen and nitrous oxide (40:60) using a Penlon AV-S ventilator (Penlon Ltd, Abingdon, UK). Minute volume was adjusted to maintain normocapnia with tidal volume set at a maximum of 8ml/kg.

Surgical sites were prepared with povidone-iodine solution (10% wt/vol, Betadine Aqueous Antiseptic Solution, Seaton Healthcare Group plc, UK).

Both femoral arteries and the left carotid artery were cannulated with a 7F Desivalve introducer sheath (Vygon, Swindon, UK) using Seldinger technique via cut down. The left jugular vein was cannulated with an 8F Portex Dog catheter (Smiths Medical, Ashford, UK) (open technique). The right jugular vein was cannulated with an 8F Desivalve introducer sheath. The right femoral vein was cannulated with a 7F 23cm Input® Introducer sheath (Medtronic Minneapolis, USA).

Pulmonary artery pressure was measured using a balloon tipped flow-directed cannula (744MF75 Swan Ganz, Edwards Life Sciences Ltd, Newbury, UK).

Following venous access: 0.9% saline (Aqupharm No1, Animalcare Ltd, York, UK) (10ml/kg) was administered until surgical preparation was complete to replace insensible losses; and surgical anaesthesia was converted from isoflurane to intravenous alphaxalone (Alfaxan® 10mg/ml single use, Jurox (UK) Ltd, Malvern Link, UK). At starting infusion of approximately 35-45ml/hr was given.

A midline laparotomy was performed to enable splenectomy, bladder cannulation with suprapubic bladder drainage catheter (BD Bonanno™ Becton, Dickinson & Co, Franklin Lakes, USA) and placement of a snare around the left medial lobe of the liver(1 Ethilon™, Ethicon®, Belgium) for later creation of uncontrolled haemorrhage. The surgical site was closed en mass to prevent hypothermia and fluid losses.

Nitrous oxide was discontinued on completion of instrumentation, and after a suitable wash-out period on enriched air, animals were weaned off the ventilator. Animals were allowed to breathe spontaneously for the remainder of the experiment unless during the Injury and Resuscitation Phases they displayed marked respiratory depression (respiratory rate <10 breaths per minute), at which stage Pressure Controlled Synchronized Intermittent Mandatory Ventilation (PC-SIMV, Drager Evita Infinity V500, Draeger Medical UK Ltd, UK) was initiated in an attempt to maintain adequate oxygenation ($SaO_2$ >90% and prevent severe hypercapnia (defined as $PaCO_2$ >7.0kPa).

*Blood product usage*

A maximum of 4 units of PRBCs and 4 units of FFP were administered to Group 3.

A maximum of 4 units of FWB were administered to Group 4

A maximum of 4 units of FFP were administered to Group 5.

Resuscitation continued with 0.9% NaCl if the maximum number of units were infused.

*Blood sample analysis*

Samples were collected at pre-defined time points throughout the study as shown in Figure 1.

Arterial and mixed venous blood gas samples were collected anaerobically into heparinised syringes from the femoral and pulmonary arterial cannulae respectively. Paired arterial and mixed venous blood gas measurements were made (ABL800 blood gas analyser, Radiometer Ltd, Crawley, UK).

Arterial blood samples were collected into EDTA vacutainers for haematology assessment (Siemens Advia 2120i Haematology Analyser, Siemens Healthcare GmbH, Germany).

*Controlled Haemorrhage Protocol*

Blood (30% original blood volume ($B_0$), Equation 1), was withdrawn manually via the femoral artery cannula at an exponentially reducing rate (Equation 2), over 9 minutes and 41 seconds.

Estimation of total blood volume using Equation 1: $B_0 = 161.4751 (W^{-0.2197})$, where $B_0$ = total blood volume (ml/kg) and W = body weight (kg).

Rate of blood loss during controlled haemorrhage using Equation 2: $V = B_0 (1-e^{-0.04t})$, where V = total blood lost at time t (ml/kg); $B_0$ = initial blood volume (ml/kg); and t = percentage time until death

*Criteria for administration of resuscitation fluid*

The trigger for initiation of fluid resuscitation was SBP 80mmHg. To account for small beat by beat fluctuations in SBP and therefore prevent 'over resuscitation' a bolus of fluid was given when SBP was in the range 75-80 mmHg for 30 seconds, or when SBP < 75 mmHg.

*Statistical analysis and power calculation*

The aim of this study was to evaluate the pathophysiology associated with prolonged hypotensive resuscitation using blood/blood products or crystalloid resuscitation in a porcine model of militarily relevant traumatic haemorrhagic shock (THS). To establish the severity of the model, the resuscitation groups were compared with a group that was not given resuscitation (No Treatment). To facilitate a power calculation during the planning phase of the study we hypothesised that the rank order of benefit would be: No treatment < Saline < Plasma < "Blood"

The rationale was that an improvement in circulating volume (Saline) would confer some benefit. However, because saline is a crystalloid (rather than a colloid) and does not contain substances, for example, beneficial to the microcirculation then plasma would be substantially better. However, since plasma would result in dilution of oxygen carrying capacity of the recipient's blood, then fluids containing red cells would be better than plasma. We were interested in two red cell containing fluids; packed red cells combined with fresh

frozen plasma (PRBC:FFP, 1:1 ratio) and fresh whole blood (FWB). The study was not designed to detect a survival difference between PRBC:FFP and FWB as this was not anticipated to be great over the time-course of the study, but we were interested in determining whether there were indications of background pathophysiological differences between PRBC:FFP and FWB. The experimental design included equal group sizes between all fluids, resulting in the "Blood" group being twice the size of the other group (to allow for two fluids). The power calculation was based on a pairwise Log Rank test focused on proportional survival to the end of the study (450 minutes), with a two sided comparison at an alpha level of rejection of P=0.05 and a minimum power of 70%. The hypothesised proportional survival rate in each group is given in the table below.

Supplemental Digital Content 1 Table 1: Hypothesised proportional survival in at the end of a 7.5h resuscitation period (simulating pre-hospital resuscitation) in four groups of animals resuscitated with the fluids indicated at the head of each column.

| **Fluid** | **No Treatment** | **Saline** | **Plasma** | **"Blood"** |
|---|---|---|---|---|
| **Proportional survival (%)** | 0 | 20 | 70 | 100 |

The number of animals required to demonstrate a significant difference for each of the planned pairwise comparison is shown in the table below.

Supplemental Digital Content 1 Table 2: Number of animals needed for each pairwise comparison shown as x/y, where the comparison is between the group indicated in the first column and the group indicated in the top row, and x is the number needed in the group indicated by the first column and y is the number needed in the group indicated in the top row. Power calculation based on a pairwise Log Rank test, with alpha level of rejection P-

0.05 and Power approximately 70%. The assumed proportional survival for each group is indicted in Supplemental Digital Content 1 Supplemental Digital Content 1 Table 1.

| | **Saline** | **Plasma** | **"Blood"** |
|---|---|---|---|
| **No treatment** | 8/8 | 3/3 | 1/4 |
| **Saline** | | 10/11 | 2/6 |
| **Plasma** | | | 10/22 |

An interim analysis was planned to determine whether it was appropriate to continue to aim for the numbers indicated in Supplemental Digital Content 1 Table 2. It became apparent that survival was substantially higher than anticipated in the saline and plasma groups, although the hypothesised rank order was preserved, with the exception of plasma, which was found to be similar to the "Blood" group. The model was clearly severe since 0% survived in the No Treatment group. The study was therefore terminated with n=9 in each group since it was clear that additional animals would not show a difference between plasma and "blood" groups. The study was therefore concluded with a planned, detailed, examination of the pathophysiological responses and outcomes, where clear differences were seen between treatment groups.
