# Supplementary material for: Resuscitation with whole blood or blood components improves survival and lessens the pathophysiological burden of trauma and haemorrhagic shock in a pre-clinical porcine model
Source: Eur J Trauma Emerg Surg. 2022 Jul 27;49(1):227–39. doi: 10.1007/s00068-022-02050-6 (PMC9925484; doi:10.1007/s00068-022-02050-6)
Supplement: Supplementary file 2 — Supplementary file2 (PDF 44 KB) [file 68_2022_2050_MOESM2_ESM.pdf]

Supplemental Digital Content 2: A between group comparison of Baseline physiological and biochemical data. Wt=body weight; SBP=systolic blood pressure; MBP=mean arterial blood pressure; DBP=diastolic blood pressure; CO=cardiac output; PaO_2=arterial partial pressure of oxygen; $PaCO_2$=arterial partial pressure of carbon dioxide; Art pH=arterial pH; DO_2=oxygen delivery; VO_2=oxygen consumption; OER=oxygen extraction ratio; ABE=arterial actual base excess; Lact = lactate; AG=Anion gap; and Hb=arterial haemoglobin

	No treatment	Saline	FFP	PRBC:FFP	FWB	P (comparison between groups)
n	9	9	9	9	9	
Wt (kg)	51.6±2.9	49.9±2.1	51.4±4.1	51.1±2.3	53.3±4.8	0.2608
SBP (mmHg)	161±13	170±10	163±18	171±14	160±22	0.6920
MBP (mmHg)	123±11	131±8	125±12	127±11	127±16	0.6331
DBP (mmHg)	103±10	112±9	105±10	106±11	106±16	0.5554
CO (l/min)	7.8±1.3	7.7±1.6	7.8±0.9	7.7±2.1	8.3±1.7	0.9456
PaO_2 (kPa)	9.8±0.8	9.5±0.8	9.3±1.1	9.5±1.0	10.2±1.1	0.3509
$PaCO_2$ (kPa)	6.6±0.4	6.7±0.7	6.6±0.7	6.3±0.8	6.4±0.5	0.6962
Art pH	7.40±0.02	7.40±0.03	7.41±0.04	7.40±0.04	7.40±0.03	0.9832
DO_2 (ml O_2/kg/min)	23.9±5.3	25.0±4.0	25.3±4.5	24.0±7.1	26.0±4.1	0.8955
VO_2 (ml O_2/kg/min)	6.7±1.8	6.4±1.4	6.5±1.4	6.2±1.7	6.2±0.9	0.9278
OER	0.28±0.04	0.26±0.05	0.26±0.03	0.27±0.07	0.24±0.03	0.3941
ABE (mM)	4.8±1.1	5.1±1.2	5.1±1.4	4.0±1.8	4.1±2.1	0.3745
Lact (mM)	1.5±0.8	1.2±0.6	1.0±0.4	1.2±0.4	2.3±1.6	0.0181
AG (mM)	7.7±1.0	7.1±2.0	7.1±1.7	7.5±2.6	8.7±1.2	0.2933
Hb (g/dl)	12.2±0.7	12.4±0.5	12.8±0.6	12.4±1.0	12.9±1.0	0.3591
