# Supplementary material for: Resuscitation with whole blood or blood components improves survival and lessens the pathophysiological burden of trauma and haemorrhagic shock in a pre-clinical porcine model
Source: Eur J Trauma Emerg Surg. 2022 Jul 27;49(1):227–39. doi: 10.1007/s00068-022-02050-6 (PMC9925484; doi:10.1007/s00068-022-02050-6)
Supplement: Supplementary file 3 — Supplementary file3 (PDF 80 KB) [file 68_2022_2050_MOESM3_ESM.pdf]

Supplemental Digital Content 3 Table 1: Statistical P values associated with differences between groups and changes over time during the shock/injury phase of the study. All groups were included in this analysis utilising a linear mixed model ANOVA with repeated measures over time, using pre-injury baseline values as the covariate. Abbreviations and units are defined in Supplemental Digital Content 2.

Variable	Differences between groups	Changes over time	Differences in pattern of response (statistical interaction)
SBP	0.6832	<0.001	0.9467
MBP	0.6924	<0.001	0.9785
CO	0.0589	<0.001	0.2841
HR	0.7977	<0.001	0.1703
SvO_2	0.2326	<0.001	0.0471
OER	0.2604	<0.001	0.2079
SaO_2	0.7631	<0.001	0.5546
DO_2	0.0236	<0.001	0.0634
VO_2	0.7301	<0.001	0.8749
ABE	0.4747	<0.001	0.1224
Lactate	0.5060	<0.001	0.1099

Supplemental Digital Content 3 Table 2: Statistical P values associated with differences in survival times between groups as indicated in the table. Survival times were compared using Kaplan-Meier plots and logrank test, followed by planned between group comparisons using Mantel-Haenszel probability levels

**Group**	**Comparison group**	**P**
Overall	All groups	0.0002
Planned pairwise comparisons		
No treatment	FWB, PRBC:FFP or FFP	0.036
No treatment	Saline	0.0404
Saline	FWB, PRBC:FFP or FFP	0.1451
Saline	**Combined** FWB **and** PRBC:FFP ("blood group")	0.0394
Comparison between FWB, PRBC:FFP and FFP		Cannot be calculated due to 100% survival in all groups

Supplemental Digital Content 3 Table 3: Statistical P values associated with differences between groups in attainment and stability of initial resuscitation during the first two hours of the resuscitation phase of the study. All groups except "No treatment" were included in this analysis. Between group comparisons of discrete data (number of boluses) were made using a Kruskal-Wallis analysis of variance, followed where appropriate by a planned between group multiple comparison assessment. Continuous data was assessed for normality and subjected to transformation if necessary, followed by 1-way ANOVA with Tukey multiple comparison of means if appropriate.

**Variable**	**Differences between groups**	**Test**
**Initiation of resuscitation and attainment of target SBP of 80 mmHg**		
Time to initiation of fluid administration (min)	0.6620	ANOVA
Number of boluses required to initially attain the target SBP of 80 mmHg	0.0052	Kruskal-Wallis
**First bolus after attaining target SBP of 80 mmHg**		
SBP before initiation of the bolus	0.1100	ANOVA
SBP 30 seconds after completion of the bolus	0.0002	ANOVA
**Next (subsequent) bolus**		
Time to bolus	0.0169	ANOVA
**Number of boluses needed to attain and then maintain target SBP of 80 mmHg**		
First hour of resuscitation	0.0001	Kruskal-Wallis
Second hour of resuscitation	0.0188	Kruskal-Wallis

Overall, the Saline group was significantly different to each of the groups given blood/blood products ($P<0.0001$ in each case), with an approximate threefold greater volume for saline. Differences between the other groups were relatively minor. The group given FFP required significantly more fluid than that given FWB ($P=0.0331$). The fluid requirement in the PRBC:FFP group was intermediate amongst the blood/blood product groups and was not significantly different to either the FFP or FWB groups ($P>0.15$ in each case).

Supplemental Digital Content 3 Table 4: Statistical P values associated with differences between groups and changes over time during the resuscitation phase of the study. All groups except "No treatment" were included in this analysis utilising a linear mixed model ANOVA with repeated measures over time, using the end-shock value as the covariate. Abbreviations and units are defined in Supplementary Table 1 with the addition of Art K^+ (arterial potassium concentration).

Variable	Differences between groups	Changes over time	Differences in pattern of response (statistical interaction)
SBP	<0.001	<0.001	0.3814
CO	0.0211	<0.001	0.0644
SvO_2	0.0002	<0.001	<0.001
SaO_2	0.2110	0.3360	0.0594
CaO2	<0.001	<0.001	<0.001
DO_2	0.1133	<0.001	0.9967
OER	0.0017	<0.001	<0.001
VO_2	0.8123	<0.001	0.5908
ABE	<0.001	<0.001	<0.001
Lactate	0.0788	<0.002	0.0029
Art pH	<0.002	<0.001	<0.001
$PaCO_2$	0.3034	<0.001	<0.005
Art K+	0.0181	<0.001	<0.001
Art Hb	<0.001	<0.001	<0.001
Cumulative resuscitation volume	<0.001	<0.001	0.4320

Biochemical changes

Saline group displayed the lowest ABE throughout the resuscitation period, and was significantly different to both FWB and FFP ($P<0.005$). At the other end of the spectrum,

FFP had the highest ABE. These changes, that were mirrored by changes in arterial pH (which also showed significant changes over time, and a difference in pattern and absolute levels between groups, Figure 5C), are likely, at least in part, to represent a metabolic acidosis. There was a partial respiratory compensation of the acidosis with significant changes in $PaCO_2$, again with different patterns between groups (Figure 5D). In addition, it is likely that there was a hyperchloraemic acidosis in the Saline group since the chloride levels were significantly elevated (Supplemental Digital content 6), and bicarbonate levels were lowest (Supplemental Digital content 6), without an increased anion gap (Supplemental Digital content 6).

The Shock Phase was associated with a rapid elevation in arterial potassium levels (K^+). There was a significant change in K^+ from the onset of resuscitation, and there were significant differences in pattern of response and absolute levels between groups, with the Saline group displaying the highest level of K^+ in the later stages of resuscitation (Supplemental Digital Content 6).
