# Supplementary figures and images for: Resuscitation with whole blood or blood components improves survival and lessens the pathophysiological burden of trauma and haemorrhagic shock in a pre-clinical porcine model
Source: Eur J Trauma Emerg Surg. 2022 Jul 27;49(1):227–39. doi: 10.1007/s00068-022-02050-6 (PMC9925484; doi:10.1007/s00068-022-02050-6)

## Online Resource 4: Time from the onset of resuscitation for initiation of first fluid bolus

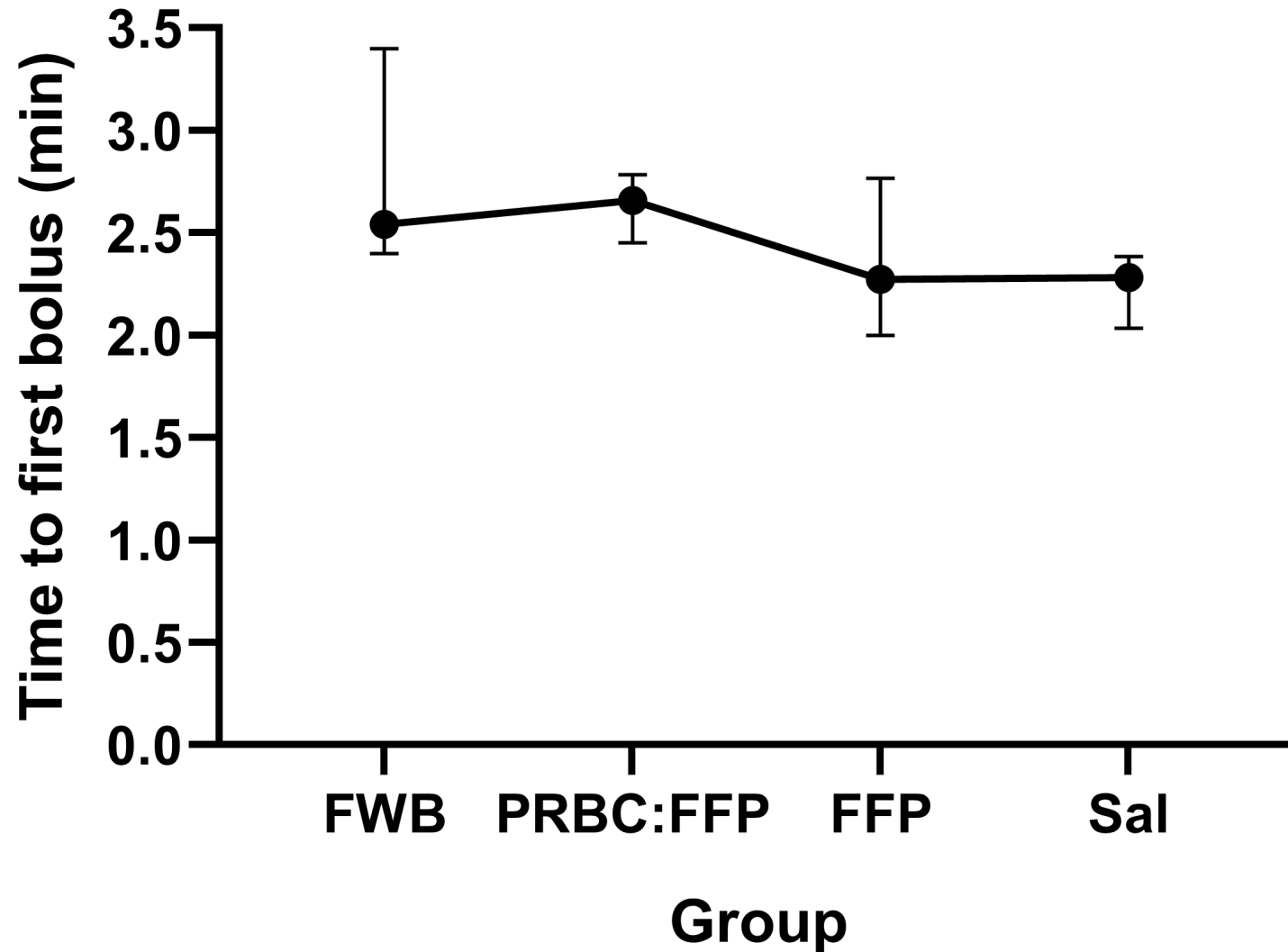

Supplement: Supplementary file 4 — Supplementary file4 (PDF 113 KB) [file 68_2022_2050_MOESM4_ESM.pdf]

## Online Resource 5: Arterial oxygen content (CaO<sub>2</sub>)

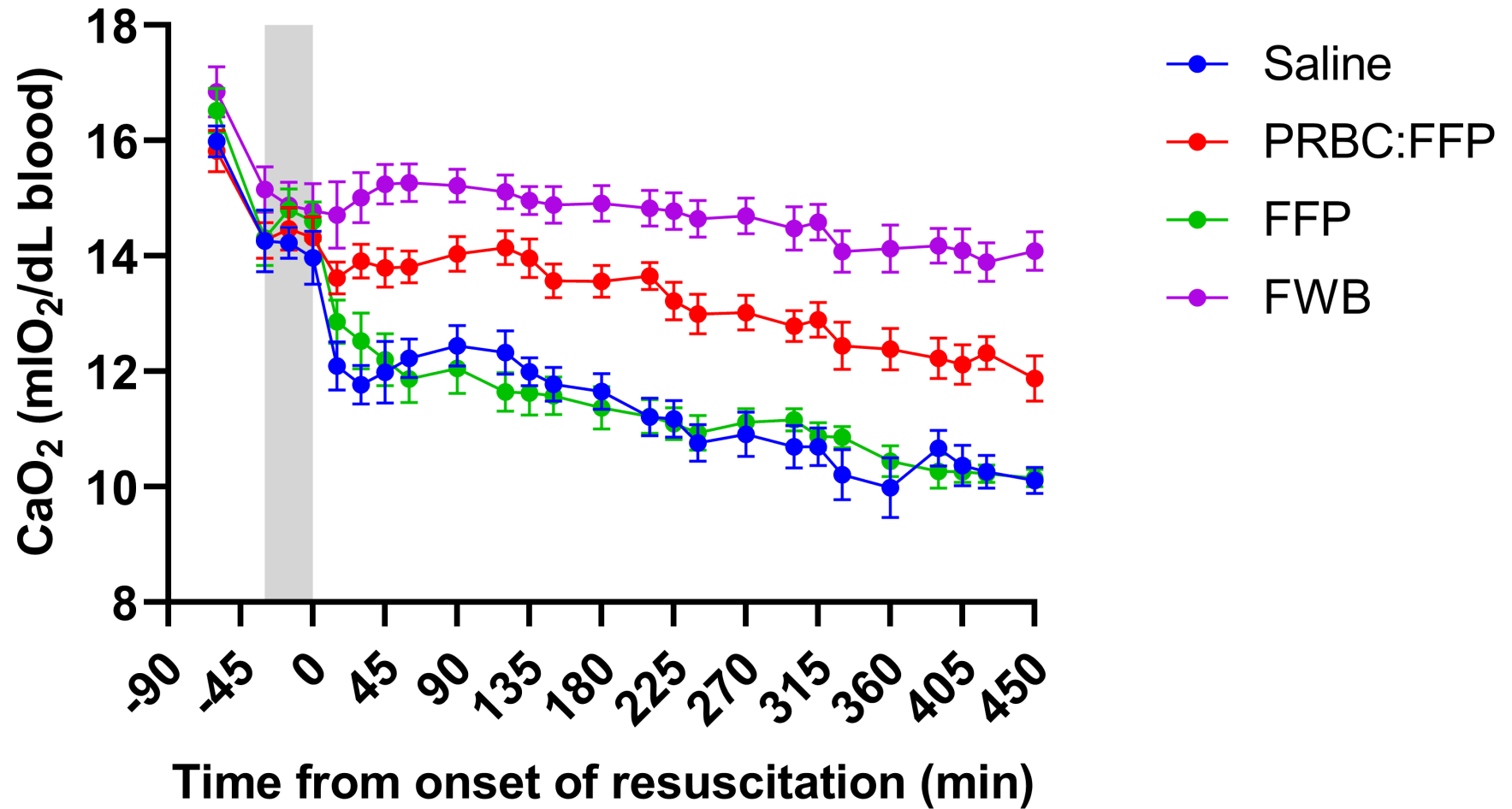

Supplement: Supplementary file 5 — Supplementary file5 (PDF 191 KB) [file 68_2022_2050_MOESM5_ESM.pdf]

# Online Resource 6: Arterial bicarbonate ( $\text{HCO}_3^-$ ), chloride, Anion Gap and potassium ( $\text{K}^+$ ) levels

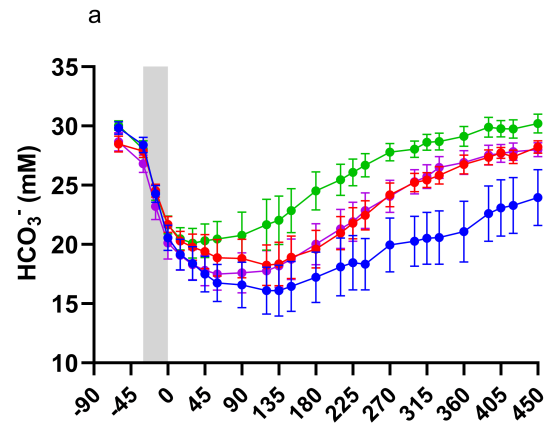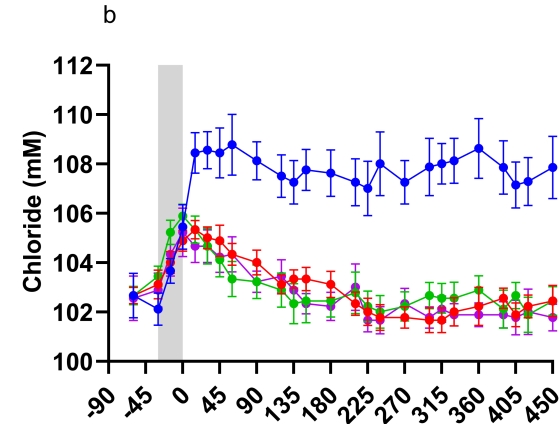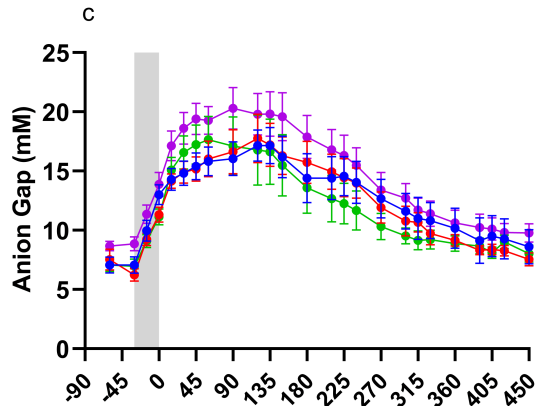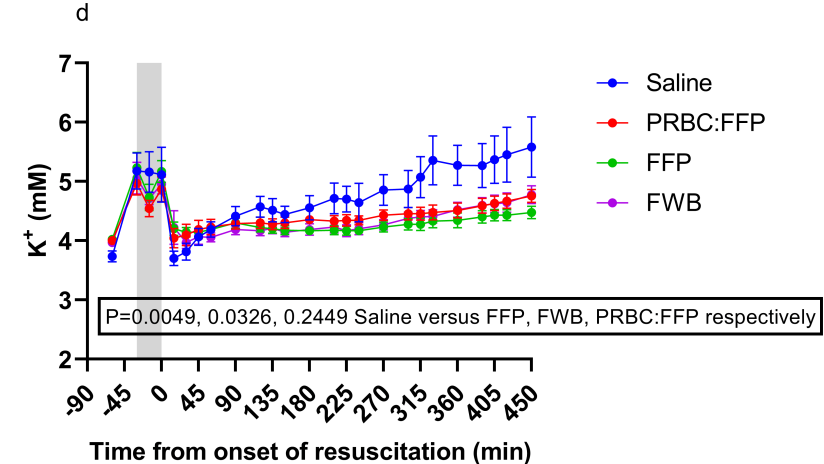

Supplement: Supplementary file 6 — Supplementary file6 (PDF 632 KB) [file 68_2022_2050_MOESM6_ESM.pdf]
