# Supplementary material for: Resuscitation with whole blood or blood components improves survival and lessens the pathophysiological burden of trauma and haemorrhagic shock in a pre-clinical porcine model
Source: Eur J Trauma Emerg Surg. 2022 Jul 27;49(1):227–39. doi: 10.1007/s00068-022-02050-6 (PMC9925484; doi:10.1007/s00068-022-02050-6)
Supplement: Supplementary file 7 — Supplementary file7 (PDF 50 KB) [file 68_2022_2050_MOESM7_ESM.pdf]

Supplementary Table 2: Volume of intra-abdominal haemorrhage determined at post-mortem examination. There were no significant differences between groups (P=0.2610). Values are mean±SEM

**Group**	**Intra-abdominal haemorrhage volume**
FWB	55±8
PRBC:FFP	59±7
FFP	73±12
Sal	186±109
No treatment	46±8

The higher volume of intra-abdominal haemorrhage (and associated standard error of the mean) in the Saline group was due to one animal in the Saline group displaying a higher haemorrhage volume. This animal had the shortest survival time within the Saline group, and the sixth shortest survival time in the study (5 No treatment animals succumbed sooner). Post-mortem examination revealed nothing unusual with respect of the liver injury, e.g. sizes and orientation of transected vessels were not different to the other animals in the study.
